# Exercise motivation: a cross-sectional analysis examining its relationships with frequency, intensity, and duration of exercise

**DOI:** 10.1186/1479-5868-7-7

**Published:** 2010-01-26

**Authors:** Lindsay R Duncan, Craig R Hall, Philip M Wilson, O Jenny

**Affiliations:** 1 School of Kinesiology, The University of Western Ontario, London, Ontario, N6A 3K7, Canada; 2 Brock University, Faculty of Applied Health Science, Department of Physical Education and Kinesiology, 500 Glenridge Avenue, St Catharines, ON, L2S 3A1, Canada; 3 Department of Kinesiology, California State University, East Bay, 25800 Carlos Bee Blvd Hayward, CA, 94542, USA

## Abstract

**Background:**

It is important to engage in regular physical activity in order to maintain a healthy lifestyle however a large portion of the population is insufficiently active. Understanding how different types of motivation contribute to exercise behavior is an important first step in identifying ways to increase exercise among individuals. The current study employs self-determination theory as a framework from which to examine how motivation contributes to various characteristics of exercise behavior.

**Methods:**

Regular exercisers (*N *= 1079; *n *= 468 males; *n *= 612 females) completed inventories which assessed the frequency, intensity, and duration with which they exercise, as well as the Behavioral Regulation in Exercise Questionnaire including four additional items assessing integrated regulation.

**Results:**

Bivariate correlations revealed that all three behavioral indices (frequency, intensity, and duration of exercise) were more highly correlated with more autonomous than controlling regulations. Regression analyses revealed that integrated and identified regulations predicted exercise frequency for males and females. Integrated regulation was found to be the only predictor of exercise duration across both genders. Finally, introjected regulation predicted exercise intensity for females only.

**Conclusions:**

These findings suggest that exercise regulations that vary in their degree of internalization can differentially predict characteristics of exercise behavior. Furthermore, in the motivational profile of a regular exerciser, integrated regulation appears to be an important determinant of exercise behavior. These results highlight the importance of assessing integrated regulation in exercise settings where the goal of understanding motivated behavior has important health implications.

## Background

Engagement in regular physical activity is an important part of a healthy lifestyle. Research has shown that regular exercise is linked to the prevention of cardio-vascular disease, type 2 diabetes, cancer, hypertension, obesity, osteoporosis, and depression [1]. The physical activity guidelines set forth by the Canadian government recommend that adults engage in a cumulative total of sixty minutes of moderate intensity physical activity (occurring in bouts of at least 10 minutes) every day [2]. In addition to this, some research has shown that vigorous exercise can lead to health benefits above and beyond those offered by moderate intensity exercise. For example, the use of antidiabetic, antihypertenstion, and LDL-C-lowering drugs have been found to have an inverse relationship with vigorous physical activity [3]. Furthermore, in a study examining the relationships between physical activity and fatness in adolescents, it was found that lower percent body fat was related to vigorous physical activity but not to moderate intensity physical activity [4]. It appears that in order to achieve the health benefits associated with physical activity it is important to exercise regularly and at an appropriate intensity. Despite the vast amount of research that has demonstrated the link between physical activity and health, 63% of Canadians are not sufficiently active to obtain these health benefits [2]. One factor that is thought to contribute to an individual's physical activity levels is his or her motivation to exercise. In fact, various types of motivation have been found to influence effort expended during exercise sessions as well as intentions to continue exercising [5].

Self-Determination Theory (SDT) [6] has been proposed as one way of looking at motivation. SDT is a general theory which has frequently been applied in the exercise domain. The SDT framework posits that human motivation lies along a continuum which represents varying degrees of autonomy. Autonomy refers to behaviors being self-determined, or freely initiated by the individual [6]. The self-determination continuum is comprised of both intrinsic and extrinsic components. Intrinsic motivation occupies the most self-determined end of the continuum and involves motivation derived from the sheer pleasure and satisfaction of engaging in the behavior itself [6]. An exerciser who is intrinsically motivated might swim, for example, because they enjoy the feeling of their body moving through the water. Four distinct behavioral regulations comprise the extrinsic part of the motivational continuum. These four regulations successively decrease in their degree of self-determination from autonomous regulations to controlling regulations. Integrated and identified regulations represent the more autonomous forms of extrinsic motivation. Integrated regulation is represented by an individual's belief that a behavior is an important part of his or her identity and is consistent with his or her personal values [7]. An individual who demonstrates integration might go running because they believe they are 'a runner' and therefore running is consistent with their sense of identity. Identified regulation refers to being motivated to perform a behavior because it is personally significant and results in outcomes which are valued by the individual [6,7]. For example, individuals might engage in resistance training because they know that weight bearing activities are important for bone health. Controlling regulations (introjected and external) occupy the less self-determined end of the motivational continuum. Introjected regulation represents the desire to obtain intrapersonal rewards (e.g., pride) or to avoid self-inflicted punishments (e.g., guilt or shame) [7] while external regulation refers to the desire to obtain external rewards or avoid punishments [7]. An individual who exercises for external reasons might do so to appease their spouse or their physician. It is also possible that an individual will be amotivated. That is, they will engage in a behavior without feeling any motivation, or they will exhibit a complete lack of intention to perform a behavior. An individual's relative location along the self-determination continuum is determined by the degree to which he or she has achieved satisfaction of the basic psychological needs for competence, autonomy, and relatedness [8].

One important contention of SDT is that the external regulations and amotivation are less adaptive in nature while intrinsic motivation results in positive motivational consequences. Research has supported this contention with amotivation being linked to behavioral disengagement and negative psychological conditions [7]. Furthermore, intrinsic motivation is associated with persistence at a task as well as psychological health and well-being [6]. In an exercise context, research has examined individuals at various stages of exercise adoption and found that individuals with tendencies toward more regular exercise are more self-determined in their motivation [9].

In spite of these findings, it has been suggested that some people may persist at sport and exercise despite being extrinsically motivated [10]. This suggestion can be highlighted by research examining the relationships between obligatory exercise and motivation. In a study involving regular exercisers it was found that individuals who are preoccupied with exercise, or who exercise at greater frequency, tend to score higher on identified regulation [11]. Furthermore, individuals who experience negative emotional consequences (i.e., anger, depression) when they miss an exercise session tend to score highly on introjected regulation. In terms of exercise intensity, for individuals who show symptoms of exercise dependence, introjected regulation approached significance as a positive predictor of strenuous exercise behavior and identified regulation was found to be a positive predictor of strenuous exercise [12].

Using SDT as a context for examining the motivation of 598 male and female university students engaged in a variety of exercise classes (e.g., weight training, aerobics, swimming), it was found that students who were classified as 'more frequent exercisers' showed higher levels of intrinsic motivation and the autonomous forms of extrinsic regulation compared to 'less frequent exercisers' [13]. A gender analysis revealed that females reported higher levels of intrinsic motivation and autonomous regulations while exercise behavior among males was more externally regulated and amotivated.

Further research examined the relationships between exercise regulations and various motivational consequences (i.e., behavioral intention, effort and importance associated with exercise participation, and current exercise behavior) among university students [5]. Results revealed that identified regulation was the strongest predictor of each of the three exercise behaviors in both males and females. Intrinsic regulation was also found to predict effort and importance for males and females, as well as behavioral intention for females only. These findings were consistent with previous research and with SDT. Interestingly, it was found that introjected regulation was a positive predictor of all three motivational consequences for females only. This finding suggested that females may experience a sense of pride associated with exercise or some degree of guilt or shame if they do not exercise.

Overall, it appears that exercise-related motivation varies according to the amount of exercise an individual undertakes. Previous research has considered exercise behavior in several different ways, from intention to exercise and self-reported exercise frequency [9] to a measure including exercise intensity [5] and indicates that different types of behavior (e.g., exercise frequency and intensity) may be differentially regulated. Since SDT suggests that the regulations along the continuum are distinct, it is possible that they can be individually manipulated. If key motivational forces can be identified in regular exercisers and specific motivational deficits can be identified in less frequent exercisers, perhaps it is possible to target the most relevant types of motivation in order to increase exercise behavior among those who are insufficiently active. If this is the case, understanding the unique role that each regulation plays in exercise behavior has important practical implications for exercise interventions.

One main limitation to research regarding motivation to exercise has been that the three basic measures of exercise behavior, frequency, intensity and duration have not been investigated within a single study. Another limitation is the lack of a measure of integrated regulation. Many of the studies examining exercise motivation have used the Behavioral Regulation in Exercise Questionnaire (BREQ) [14] which does not measure integration. More recently, a measure of integrated regulation which is complementary to the BREQ has been developed [15]. The inclusion of an integrated subscale allows for the full spectrum of motives to be measured which is important in order to gain a complete understanding of how individuals are motivated to engage in exercise. Therefore, the purpose of this study was to examine the relationships between three exercise behaviors (frequency, intensity, and duration) and the various behavioral regulations according to the SDT framework, including integrated regulation. Based on the contention of SDT that free-choice behaviors are most closely related to more self-determined motives, it was hypothesized that all three exercise behaviors would be most closely related to autonomous regulations and intrinsic motivation.

## Methods

### Participants

Participants (*N *= 1054) were male (*n *= 460) and female (*n *= 594) volunteer (*M*_*age *_= 24.15, *SD *= 9.61) regular exercisers. For the purposes of this study, 'regular exercise' was defined as consistently engaging in at least two exercise sessions (of any kind) each week for the past six months. The sample was largely composed of students with 75% of participants reporting 'student' as their primary occupation. Self-report data revealed the sample was quite active (*M*_frequency _= 4.07 sessions per week, *SD *= 1.77; *M*_duration _= 67.31 minutes per session, *SD *= 28.23; and *M*_intensity _= 69.71 weekly METS, *SD *= 39.65). Participants listed the exercise activities in which they typically participate. The most commonly cited exercise activities were running (62.6%), weight training (61.2%), playing sports (58.7%), walking (48.5%), and exercising on cardio equipment (e.g., treadmills, stationary bikes, elliptical trainers; 46.8%).

### Measures

Exercise behavior was assessed using a self-report measure in which participants indicated the number of times they exercise in a typical week, the average duration of each session, and the type of exercises they engage in.

The Leisure Time Exercise Questionnaire (LTEQ) [16] was used to assess participants self-reported exercise intensity. Participants indicated the frequency of mild, moderate, and strenuous activity they engage in for at least 15 minutes during a typical week. A composite exercise behavior score was then calculated using the weighted sum of each exercise intensity according to the following formula: (mild × 3) + (moderate × 5) + (strenuous × 9). The result was a weekly MET (units of metabolic equivalence) value. The LTEQ has been found to be valid and reliable when compared to objective measures of physical activity [17].

The Behavioral Regulation in Exercise Questionnaire-version 2 (BREQ-2) [18] is a 19-item self-report measure which was adapted from the original BREQ [14] and assesses exercise regulations according to the SDT framework. The BREQ-2 includes 5 subscales assessing intrinsic (e.g., "I enjoy my exercise sessions;" *n *= 4), identified (e.g., "It's important to me to exercise regularly;" *n *= 4), introjected (e.g., "I feel guilty when I don't exercise;" *n *= 3), and external (e.g., "I feel under pressure from my family/friends to exercise;" *n *= 4) regulations as well as amotiviation (e.g., "I don't see why I should have to exercise;" *n *= 4). Each item is rated on a 5-point scale ranging from 0 = "not true for me" to 4 = "very true for me." Recently, the BREQ-2 has been extended to include four additional items assessing integrated regulation (e.g., "I exercise because it is consistent with my values;" *n *= 4) [15]. The integrated subscale was included in the current study. A reliability analysis revealed internal consistency values ranging from .76 to .90 for the various regulations for males and females, with the exception of amotivation for males being .54 (see Table 1 for specific values).

**Table 1:** Descriptive statistics for age, frequency, duration, intensity and BREQ subscales

Variables		Females (*n *= 594)			Males (*n *= 460)		
		*M*	*SD*	*α*	*M*	*SD*	
Age		24.15	9.81		24.15	9.35	
Frequency (times/week)		3.97	1.70		4.20	1.84	
Duration (mins)		65.21	26.80		70.02	29.78	
Intensity (LTEQ-METS)		70.55	38.42		68.63	41.19	
Intrinsic motivation		3.06	0.75	.87	3.07	0.82	.88
Integrated regulation		2.70	1.02	.90	2.76	1.00	.89
Identified regulation		3.22	0.68	.76	3.16	0.74	.79
Introjected regulation		1.97	1.08	.80	1.72	1.15	.82
External regulation		0.82	0.84	.84	0.84	0.87	.85
Amotivation		0.13	0.31	.54	0.20	0.47	.79

Note. *M *= Mean; SD = standard deviation; BREQ = Behavioral Regulation in Exercise Questionnaire; LTEQ = Leisure Time Exercise Questionnaire; BREQ scale, 0 = "not true for me" to 4 = "very true for me.

### Procedures

All study procedures were approved by a university research ethics board. Participants were approached by the researcher prior to or following their workouts in their regular gym setting. Once informed consent was obtained, the participants completed the BREQ-2R, LTEQ, and demographic information.

## Results

### Preliminary Data Screening

The data was screened in order to detect missing values and outliers as well as to test for conformity with the assumptions of multiple regression (normality, linearity, and homoscedasticity). The analysis revealed no missing values and no cases of extreme responses (values > 4 standard deviation units from the mean on any measure). An examination of the distribution properties and histograms of each variable indicated that the amotivation variable deviated substantially from normality and warranted transformation. A logarithmic transformation with the addition of a constant to each value was conducted in order to normalize the amotivation variable [19]. All subsequent analyses were conducted using the transformed amotivation variable. No violations to the assumptions of linearity or homoscedasticity were observable through an examination of scatterplots of the residuals indicating that the data was suitable to undergo regression analyses.

### Descriptive Statistics, T-tests, and Bivariate Correlations

Descriptive statistics for both males (*n *= 460) and females (*n *= 594) are presented in Table 1. The data confirmed that the males and females were all highly active, reporting mean exercise frequency scores of 4.20 (*SD *= 1.84) and 3.97 (*SD *= 1.70) workouts per week respectively. Furthermore, mean scores for the exercise intensity variable (weekly METS) were slightly higher than values reported in previous research [5,20].

Mean scores for the subscales of the BREQ revealed an expected pattern in which individuals reported participating in exercise for more autonomous reasons compared to more controlling reasons [5,21]. Specifically, for both genders, identified was the most strongly endorsed regulation followed by intrinsic, integrated, introjected, external, and amotivation respectively.

T-tests revealed that males and females differed significantly (*p *< .017 to control for type 1 error) in terms of the typical duration of exercise sessions (*t*(931.79) = 2.72, *p *= .007). In this case, males reported exercising for longer durations than females (Table 1). No significant difference was observed between males and females number of exercise sessions per week (frequency; *t*(1052) = 2.07, *p *= .039) or for exercise intensity (*t*(1052) = -.78, *p *= .436).

Correlations were conducted between each of the variables of the BREQ and the three exercise behaviors (frequency, intensity, and duration; Table 2 and Table 3). The analyses revealed a theoretically consistent pattern of relationships in which adjacent subscales from the BREQ were more strongly and positively correlated with subscales theorized to be more proximal along the motivation continuum. This finding is consistent with previous research [5] and supports the concept of a motivational continuum as proposed by SDT [6-8].

**Table 2:** Bivariate Correlations between BREQ, exercise frequency, duration, intensity for males

Variable		1	2	3	4	5	6	7	8	9
1. Frequency		-								
2. Duration		.26**	--							
3. Intensity		.45**	.19**	-						
4. Intrinsic motivation		.30**	.24**	.20**	-					
5. Integrated regulation		.41***	.30***	.21***	.65***	-				
6. Identified regulation		.42***	.29***	.22***	.65***	.78***	-			
7. Introjected regulation		.25**	.23**	.17**	.32**	.50***	.53***	-		
8. External regulation		.03	.03	.07	-.08	.09	.05	.35**	-	
9. Amotivation		-.20**	-.06	-.14	-.33**	-.35***	-.44***	-.13**	.25**	-

**p < .01.

*** *p *< .001.

**Table 3:** Bivariate Correlations between BREQ, exercise frequency, duration, intensity for females

Variable		1	2	3	4	5	6	7	8	9
1. Frequency		-								
2. Duration		.31**	--							
3. Intensity		.43**	.22**	-						
4. Intrinsic		.34**	.18**	.25**	-					
5. Integrated regulation		.41***	.30***	.21***	.65***	-				
6. Identified regulation		.42***	.29***	.22***	.65***	.78***	-			
7. Introjected regulation		.24**	.12**	.20**	.21**	.50***	.53***	-		
8. External regulation		-.09*	-.06	-.01	-.16**	.09	.05	.27**	-	
9. Amotivation		-.14**	-.12**	-.16**	-.28**	-.35***	-.44***	-.10**	.25**	-

**p < .01.

*** *p *< .001.

Strong correlations were found between the identified and integrated subscales for both males (*r *= .74, *p *= .0001) and females (*r *= .78, *p *= .0001). Some researchers have indicated that bivariate correlations >.70 between variables may suggest collinearity [19]. An examination of the collinearity diagnostics revealed that when the condition index was high (>10), no two variables had variance proportions exceeding the recommended threshold (.50) [22] and therefore it was determined that the subscales were not collinear.

In line with self-determination theory, all three exercise behaviors were more strongly correlated with intrinsic motivation and the more autonomous forms of extrinsic motivation for males and females. For males, exercise frequency and intensity were most strongly related to identified regulation, while duration of exercise was most strongly related to integrated regulation. Similar to males, for females, identified regulation had the strongest relationship with exercise intensity and integrated regulation was most strongly related to frequency. For females, however, integrated regulation was also most strongly related to duration of exercise.

### Simultaneous Multiple Regression Analyses

Regression analyses were conducted to examine the relationships between exercise regulations and the three exercise behaviors. Based on the results of the *t*-tests revealing a significant difference between males and females for one of the dependent variables (i.e., duration of exercise), and previous research which has conducted regression analyses separately by gender [5], the current analyses involved separate regression analyses for males and females.

Results of the analyses revealed that integrated and identified regulations were significant predictors of exercise frequency for both males and females (Table 4). In terms of duration of exercise, integrated regulation was found to be a significant and positive predictor for males and females (Table 5). Finally, introjected regulation was found to be a positive predictor of exercise intensity for females only, while none of the behavioral regulations were a unique predictor of intensity among men (Table 6).

**Table 4:** Multiple regression analysis predicting exercise frequency from exercise regulations

Variable		*F*	*df*	*R^2adj^*	B	SE B	*β*	*t*
Males		18.25***	6, 453	.18				
Intrinsic motivation					-.02	.13	-.01	-.16
Integrated regulation					.38	.13	.20	2.82**
Identified regulation					.62	.19	.25	3.23**
Introjected regulation					.04	.09	.02	.42
External regulation					-.01	.10	-.01	-.13
Amotivation					-.22	.75	-.02	-.30
Females		38.50***	6, 587	.28				
Intrinsic motivation					-.03	.11	-.02	-.30
Integrated regulation					.65	.09	.39	7.20***
Identified regulation					.38	.15	.15	2.51**
Introjected regulation					.09	.07	.06	1.35
External regulation					-.15	.08	-.07	-1.92
Amotivation					.17	.71	.01	.23

*p < .05.

** *p *< .01.

*** *p *< .001.

**Table 5:** Multiple regression analysis predicting exercise duration from exercise regulations

Variable		*F*	*df*	*R^2adj^*	B	SE B	*β*	*t*
Males		9.48***	6, 453	.10				
Intrinsic motivation					2.11	2.26	.06	.93
Integrated regulation					4.80	2.26	.16	2.13*
Identified regulation					4.69	3.26	.12	1.44
Introjected regulation					2.44	1.49	.09	1.64
External regulation					-1.30	1.73	-.04	-.75
Amotivation					22.24	12.75	.09	1.74
Females		6.61***	6, 587	.05				
Intrinsic motivation					2.80	2.01	.08	1.39
Integrated regulation					4.98	1.61	.19	3.09**
Identified regulation					-2.84	2.74	-.07	-1.04
Introjected regulation					1.77	1.18	.07	1.50
External regulation					-1.58	1.40	-.05	-1.13
Amotivation					-18.29	12.68	-.06	-1.44

*p < .05.

** *p *< .01.

*** *p *< .001

**Table 6:** Multiple regression analysis predicting exercise intensity from exercise regulations

Variable		*F*	*df*	*R^2adj^*	B	SE B	*β*	*t*
Males		5.28***	6, 453	.05				
Intrinsic motivation					4.54	3.12	.09	1.42
Integrated regulation					1.51	3.20	.04	.47
Identified regulation					3.68	4.63	.07	.79
Introjected regulation					1.85	2.11	.05	.88
External regulation					3.33	2.45	.07	1.36
Amotivation					-28.20	18.09	-.08	-1.56
Females		11.22***	6, 587	.09				
Intrinsic motivation					5.47	2.82	.11	1.94
Integrated regulation					3.02	2.26	.08	1.34
Identified regulation					4.42	3.84	.08	1.15
Introjected regulation					3.85	1.66	.11	2.32*
External regulation					.40	1.97	.01	.20
Amotivation					-32.11	17.78	-.08	-1.81

*p < .05.

** *p *< .01.

*** *p *< .001

## Discussion

The purpose of this study was to examine the relationships between three exercise behaviors (frequency, intensity, and duration) and various behavioral regulations. For males and females, both identified and integrated regulations were found to be positive predictors of exercise frequency. These findings are consistent with our original hypothesis and with the contention of SDT that free choice behaviors can be predicted by more autonomous motives. Previous research examining the relationship between behavioral regulations and various motivational consequences (i.e., behavioral intention, effort and importance, and exercise behavior) found that more autonomous regulations predicted behavioral intention however integrated regulation was not assessed [5]. These findings highlight the importance of including a measure of integrated regulation when assessing exercise motivation.

In the current investigation, integrated regulation was the strongest (and the only significant) predictor of exercise duration for males and females. This finding suggests that individuals are more likely to engage in longer bouts of physical activity if they feel that exercising is consistent with their identity. If free choice behaviors, such as exercise, are associated with autonomous motivation in general, why did integrated regulation emerge as the only significant predictor of exercise duration? Perhaps it is the nature of the exercise behavior under investigation. Without a doubt, individuals who are characterized as regular exercisers are aware of the many physiological and psychological benefits that are associated with routine (i.e., regular frequency) exercise. As a result, it is not surprising that regular exercisers have aligned their values and goals with routine exercise. It also seems logical that individuals who value the benefits associated with regular exercise have incorporated that behavior into their sense of identity. With exercise frequency, more is generally thought of as better, but with exercise duration, longer is not necessarily better. The duration of exercise depends on the goals of the exercise program and the intensity of the exercise and it can range from a few minutes to a few hours [23]. As a result, individuals may not build their exercise-related values and goals based on a 'longer is better' conception. It is possible however, that longer bouts of exercise play a stronger role in confirming ones exercise identity compared to shorter bouts of exercise, in part, as a function of the amount of time the individual invests each week in exercise participation.

The results of the current study revealed the importance of integrated regulation in the prediction of regular physical activity. Integration involves identifying that engaging in a behavior is an important part of one's identity, is proposed to be the most autonomous of the external regulations, and tends to be associated with behavioral persistence and more adaptive psychological outcomes [24]. The current analyses revealed that, in the motivational profile of a regular exerciser, the creation of an identity surrounding exercise, that is, believing that being 'an exerciser' is an important part of 'who I am' is crucial. In light of this, exercise practitioners should work to develop programs which seek to integrate exercise into an individual's personal value system and help to influence people to include the word 'exerciser' as a self-descriptor. For individuals who are moderately active, it may be possible to influence integrated regulation through a goal setting intervention in which goal setting could be used as a conduit for enhancing exercise-related identity. For example, the exerciser could be encouraged to establish exercise-related goals and use self-monitoring to track their progress toward these goals. In turn, this self-monitoring could be used as a means to substantiate the individual's exercise identity.

Interestingly, introjected regulation was the only significant predictor of exercise intensity, and this was the case for females only. Exercise intensity was not predicted by the autonomous regulations or intrinsic motivation. This finding is consistent with previous findings that introjected regulation predicted exercise behavior and the effort and importance associated with exercise for females [5] and suggests that intense exercise is driven by a sense of obligation, rather than more adaptive and personally significant motives. Research examining the motivating forces behind exercise dependence has found a similar link. For example, it has been found that introjected regulation was the strongest predictor of exercise dependence [25]. In addition, researchers examined individuals who exhibited symptoms of exercise dependence and found that introjected regulation approached significance as a positive predictor of exercise intensity [12].

It is not surprising that a sense of obligation drives exercise intensity for women. This may even be the case for women who are not exercise dependent. One study found that college-aged women believe that intense physical activity expends more energy than longer sessions of lower intensity physical activity (such as household chores) although this is not necessarily the case [26]. This study also found that college-aged women focused on the rate of caloric expenditure as an indication of an effective activity. Further research found that throughout an acute exercise session, women experienced a significant decrease in positive affect between the first minute of exercise and the minute before they reached their ventilatory threshold [27]. Together, these findings demonstrate that while women feel the need to exercise intensely, they do not tend to enjoy this type of activity. The problem that arises with this is that people who experience more controlling types of behavioral regulation tend not to persist at an activity for extended periods of time [28,29]. While women recognize the importance of more intense exercise, they are not exhibiting motivation that will lead them to persist at higher intensity exercise. Exercise programs and interventions that are autonomy enhancing may prove to be effective in increasing the frequency with which women engage in intense exercise.

While this study did provide some insight into the link between motivation and exercise behavior, the results must be interpreted with some degree of caution as all of the exercise behavior measures were taken by self-report. Specifically, it is difficult to accurately assess exercise intensity using self-report measures [30]. Future research would do well to examine the link between motivation and objectively measured exercise behavior. Further, the current sample was composed primarily of undergraduate students. While this sample did allow for the depiction of the motivational profile of a regular exerciser, it may be specific to the sample under investigation. For example, the motivational drive and exercise-related identity of a middle-aged male is likely to be strikingly different from that of a university-aged female. Over the lifespan, the role that exercise plays in an individual's life is likely to change. In the current investigation it was noted that males and females differed in terms of the role that introjected regulation played in motivating regular exercise behavior. Perhaps this difference would be attenuated (or enhanced) for males and females in different age groups. While general motivational patterns are likely to remain constant (e.g., exercisers experiencing primarily by autonomous motives), it may be more realistic to examine motivational profiles that are specific to different demographic groups.

There are two important strengths associated with this study. First, we were able to measure the entire spectrum of motives proposed by SDT [6]. This allowed us to identify the most important motivational forces behind various characteristics of exercise behavior. Second, we examined the motivation behind three different characteristics of exercise in the same study. This allowed us to gain some understanding about how motivation affects decisions to engage in exercise of varying frequency, intensity, and duration. It may be possible to influence persistence among exercise initiates using interventions that target their motivational profile and influence their development toward a profile more similar to that of a regular exerciser. Overall, the current investigation provides an important first step in determining the motivational profile of a regular exerciser which will provide a necessary point of reference with which to develop such interventions.

## Conclusions

The results of the present study revealed that various characteristics of exercise are differentially regulated. Furthermore, in the motivational profile of a regular exerciser, identified and integrated regulations are important contributors to exercise frequency while integrated regulation plays an important role in determining exercise duration. The influence that integration has on exercise behavior indicates that an individual's exercise-related identity can be influential in determining their exercise behavior. This finding points to the importance of measuring integrated regulation in an exercise context and the need for practitioners to develop programs that aim to enhance exercise-related identity in order to increase exercise participation among individuals.

## Competing interests

The authors declare that they have no competing interests.

## Authors' contributions

LRD has contributed to the collection, analysis, and interpretation of data as well as drafting the manuscript. CRH has contributed to the interpretation of the data and revisions of the manuscript. PMW has contributed to drafting the background information, analyzing the data and critically revising the manuscript. JO has contributed to the data collection as well as drafting and revising the manuscript. All authors have given final approval of the version to be published.

## Authors' information

LRD - MA, PhD Candidate, School of Kinesiology, The University of Western Ontario, Canada.

CRH- PhD, Professor, School of Kinesiology, The University of Western Ontario, Canada.

PMW- PhD, Associate Professor, Faculty of Applied Health Science, Department of Physical Education and Kinesiology, Brock University, Canada.

JO- PhD, Lecturer, Department of Kinesiology, California State University, East Bay, United States.
